# Mosaic chromosomal alterations are suppressed in older adults with HIV

**DOI:** 10.1038/s42003-026-10532-1

**Published:** 2026-07-21

**Authors:** Kiarash Behrouzfar, Win Min Han, Rashindrie Perera, Jason Li, Katherine E. Scull, Kerryn Howlett, Mark Bloch, David A. Baker, Beng Eu, Ellen Bowden-Reid, Don E. Smith, Jennifer F. Hoy, Ian Woolley, Robert Finlayson, David J. Templeton, Gail V. Matthews, Jane Costello, Mark A. Dawson, Sarah-Jane Dawson, Mark N. Polizzotto, Kathy Petoumenos, Nila J. Dharan, Paul Yeh

**Affiliations:** 1 Department of Medicine, School of Clinical Sciences at Monash Health, Monash University, Clayton, VIC Australia; 2 Kirby Institute, University of New South Wales, Sydney, NSW Australia; 3 School of Electrical, Mechanical and Infrastructure Engineering, University of Melbourne, Melbourne, VIC Australia; 4 Division of Cancer Research, Peter MacCallum Cancer Centre, Melbourne, VIC Australia; 5 Holdsworth House Medical Practice, Sydney, NSW Australia; 6 East Sydney Doctors, Sydney, NSW Australia; 7 Prahran Market Clinic, Melbourne, VIC Australia; 8 Melbourne Medical School, University of Melbourne, Melbourne, VIC Australia; 9 Albion Centre, South Eastern Sydney Local Health District, Sydney, NSW Australia; 10 School of Population Health, University of New South Wales, Sydney, NSW Australia; 11 Department of Infectious Diseases, School of Translational Medicine, The Alfred Hospital and Monash University, Melbourne, VIC Australia; 12 Centre for Inflammatory Diseases, Monash University, Clayton, VIC Australia; 13 Taylor Square Private Clinic, Darlinghurst, NSW Australia; 14 Department of Sexual Health Medicine and Sexual Assault Medical Service, Sydney Local Health District, Sydney, NSW Australia; 15 Discipline of Medicine, Central Clinical School, Faculty of Medicine and Health, The University of Sydney, Sydney, NSW Australia; 16 St Vincent’s Hospital, Darlinghurst, NSW Australia; 17 Positive Life, Sydney, NSW Australia; 18 Peter MacCallum Cancer Centre, Melbourne, VIC Australia; 19 Sir Peter MacCallum Department of Oncology, University of Melbourne, Melbourne, VIC Australia; 20 Collaborative Centre for Genomic Cancer Medicine, University of Melbourne, Melbourne, VIC Australia; 21 Clinical Hub for Interventional Research, Australian National University, Canberra, ACT Australia; 22 Monash Haematology, Monash Health, Clayton, VIC Australia

**Keywords:** Translational research, Haematopoietic stem cells, HIV infections, Predictive markers

## Abstract

Somatic gene mutations (SGMs) that drive clonal haematopoiesis (CH) have been shown to be more prevalent in people with HIV (PWH), potentially contributing to the higher risk of comorbidities in PWH compared to those without HIV. It is unknown whether mosaic chromosomal alterations (mCA), another form of CH, are associated with HIV. We demonstrate, for the first time, a markedly lower prevalence of mosaic chromosomal alterations (mCA), particularly loss of chromosome Y, in PWH compared to participants without HIV - an opposing pattern to SGMs. Our findings that mCA development may be suppressed in HIV infection suggest that the selective pressures driving mCA-related CH differ fundamentally from those driving SGM-related CH.

## Abstract (web summary)

The prevalence of Clonal Haematopoiesis (CH) driven by mosaic chromosomal alterations is lower in people with HIV in comparison with people without HIV, which contrasts with the higher prevalence of CH driven by SGMs in people with HIV.

## Introduction

Antiretroviral therapy (ART) has significantly improved the life expectancy for people with HIV (PWH)^1^. Despite these advances, PWH, including those with virological suppression on ART^2,3^ remain at increased risk of age-related comorbidities, including cardiovascular diseases and non-AIDS defining malignancies, compared to people without HIV^4–7^. These studies suggest that the pathophysiological mechanisms associated with HIV infection or ART may predispose PWH to these comorbidities.

Clonal haematopoiesis (CH) is a condition characterised by the clonal expansion of haematopoietic stem cells^8^ that is driven by genetic alterations and associated with chronic HIV infection^9^. Genetic alterations driving CH can occur at the gene level through somatic gene mutations (SGMs) or at the chromosomal level through large mosaic chromosomal alterations (mCAs) and can confer a selective clonal advantage to haematopoietic stem cells^10–12^. Several studies in the general populations have shown that these genetic alterations are associated with an increased risk of age-related diseases^11–15^, many of which are comorbidities that are either more prevalent^16^ or more likely to occur in PWH than in people without HIV^17^.

In the Age-related Clonal Haematopoiesis in an HIV Evaluation Cohort (ARCHIVE), we showed that SGMs (i.e., single nucleotide variants or small insertions/deletions) driving CH are more frequent in PWH compared to participants without HIV and are associated with geriatric syndromes such as reduced quality of life and frailty^9,18^. However, the relationship between mCA and HIV infection remains unexplored. Given the potential distinction in clonal selection and comorbidity risks between mCA, particularly mosaic loss of chromosome Y (mLOY), and SGMs^11,19,20^, assessing the relationships between these genetic alterations driving CH and HIV infection is important for understanding the biological and clinical significance of CH in PWH. Therefore, we sought to assess the relationship between mCA prevalence, HIV infection and SGM occurrence, to determine whether mCA-driven CH reflects a unique HIV-associated risk factor of age-related diseases in PWH.

## Results and discussion

**Fig. 1:**
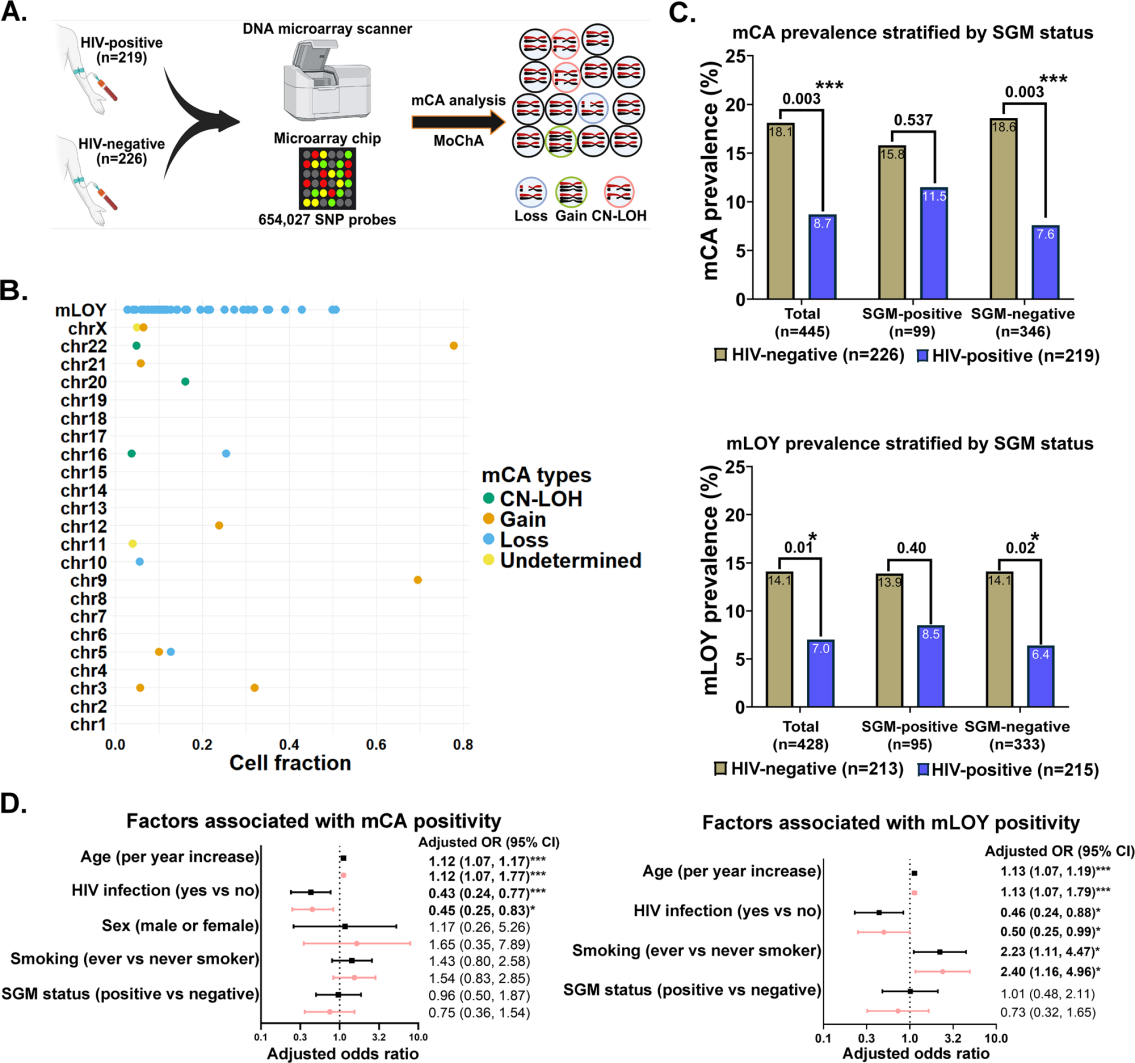
Distribution of mosaic chromosomal alterations (mCAs) and somatic gene mutations (SGMs) in ARCHIVE study (*n* = 445). **A** Overview of the experimental design and the analysis method of the study. Created in BioRender. Behrouzfar, K. (2026) https://BioRender.com/m56zp6p. **B** Visualisation of autosomal and non-autosomal mCA detected across the chromosomes. Each dot point represents a mCA event that is carried by an individual. Colours of dot points refer to mCA types i.e., gain, loss and Copy Number-Loss Of Heterogeneity (CN-LOH). **C** mCA and mosaic loss of chromosome Y (mLOY) prevalence comparison between participants without HIV (HIV-negative) and people with HIV; PWH (HIV-positive) across all participants and participants with and without SGMs. *P* values of chi-squared test for each comparison are shown on top of each line. Asterisk indicates *P* < 0.05. **D** Forest plots show logistic regression analysis outputs for mCA and mLOY occurrence (positivity) and clinical data of all participants in ARCHIVE cohort. Univariate and multivariate adjusted odds ratios with the 95% confidence intervals are shown in pink and black horizontal lines, respectively. The mean odds ratio and 95% confidence interval values are shown in the right-end column of the table. *P < 0.05, **P < 0.01 and ***P < 0.001.

To investigate whether HIV infection is associated with mCA occurrence in whole genome, we leveraged MOsaic CHromosomal Alteration (MoChA) caller to detect and compare mCA prevalence between the ARCHIVE participants with and without HIV (Fig. 1A). The ARCHIVE participants were predominately male (96.2%), with a median age of 63 (interquartile range (IQR) = 59–69) years and most participants were men who have sex with men (87.4%). The demographic and clinical characteristics of the cohort were described previously^9^. Our analysis demonstrated that 60 (13.5%) of the 445 individuals had either autosomal or non-autosomal mCA events (supplementary Table 1). While up to two mCA events were detected in chromosome 3, 5, 16 and 22, mLOY was the most frequent event, accounting for 74% of all mCAs in the study population (Fig. 1B, Supplementary Fig. 1). Among mCA events detected in HIV-positive participants in our cohort, mLOY, chr5q loss and chr21q gain were also reported in a recent preprint from the Swiss HIV Cohort Study^21^. In addition, mLOY^20,22^, chr20q loss^11,23^ and chr16q loss^11^ have been detected in studies of general populations and were associated with an increased risk of incident blood cancers, blood cell count abnormalities and cardiovascular diseases. These observations support a potential link between mCA identified in this study and the increased risk of comorbidities in PWH.

Strikingly, our analysis demonstrated that mCAs were significantly less prevalent in PWH (HIV-positive participants) compared to HIV-negative participants (8.7% vs 18.1%, respectively. *p* = 0.003, Fig. 1C). Our results differ from those of the Multicenter AIDS Cohort Study (MACS), which showed similar mCA prevalence between men living with and without HIV^24^. However, the MACS population was considerably younger (only 18.6% PWH were older than 55 years) compared to the ARCHIVE population where all participants were above 55 years, suggesting that the negative association between mCA occurrence and HIV may emerge at older ages in PWH.

Multivariable analysis demonstrated that while mCA prevalence was associated with age, the negative association between mCA and HIV was independent of age, sex, smoking history and SGM status (Fig. 1D). This suggests that the lower mCA prevalence in PWH may reflect a distinct relationship that is not explained by age alone. We also found no significant difference in average mCA clone size (cell fraction%) i.e., the proportion of blood cells harbouring mCA, or in the rate of age-associated increase in mCA clone size between HIV-positive and HIV-negative participants (Supplementary Fig. 2A–B). Given that the accumulating evidence, including the ARCHIVE study, previously revealed a higher prevalence of SGMs in older PWH compared to participants without HIV^9,25,26^, these findings highlight a potential divergence in biological processes favouring the expansion of cell clones harbouring SGMs compared to those with mCA in PWH.

**Fig. 2:**
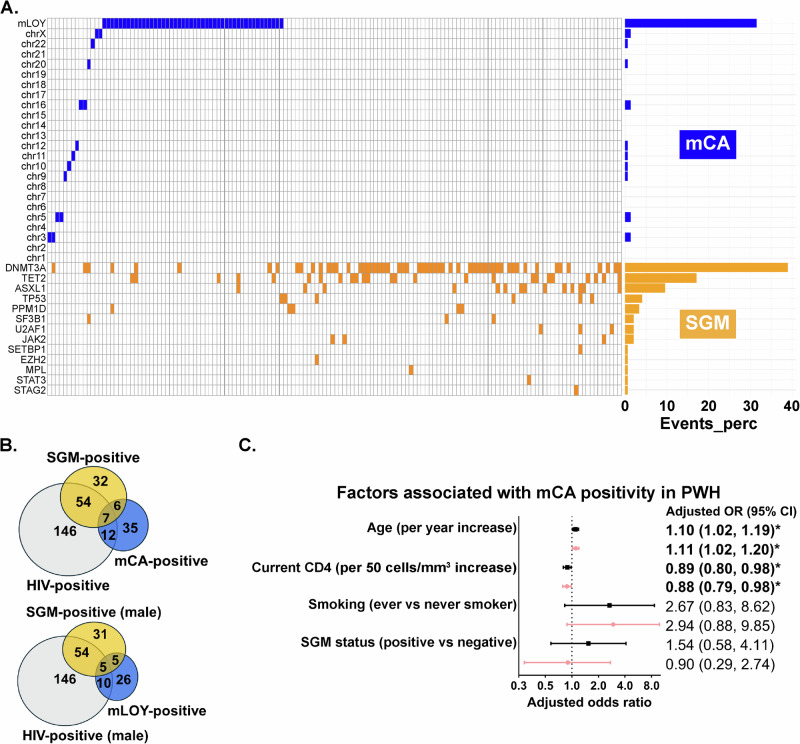
Summary of the mosaic chromosomal alterations (mCA) occurrence across ARCHIVE participants with or without somatic gene mutations (SGMs) and its associations with CD4 + T cells and clinical variables in people with HIV (PWH). **A** Heatmap summarising the distribution of participants with mosaic chromosomal alterations (mCAs) or SGMs. Each column corresponds to a participant with mCA (i.e., autosomal chromosomal alterations and mosaic loss of sex chromosomes) or SGMs and each filled square represents the presence of mCA or the respective SGMs in an individual.** B** Venn diagrams showing the number of people living with HIV (PWH) harbouring either SGMs or mCA or mosaic loss of chromosome Y (mLOY) and co-occurrence of all events. **C** Logistic regression analysis outputs for mCA positivity and clinical variables of PWH. The 95% confidence intervals are shown in the right-end column of the table. Current CD4 refers to the measurements of CD4 level in PWH at the time of sample collection and it was categorised into groups of individuals per 50 cells/mm^3^ increase. *P < 0.05 and **P≤ 0.01 ***P ≤ 0.001.

To further explore the interplay between mCA and SGM, we assessed the co-occurrence of mCAs with SGMs. In the ARCHIVE cohort, SGMs occurred in 99 of 445 participants, with *DNMT3A*, *TET2* and *ASXL1* being the most frequently mutated genes (Fig. 2A). However, we did not find a statistical association between SGMs and mCAs among all participants or within participants with or without HIV in the ARCHIVE cohort (Supplementary Fig. 3A–C). Moreover, we found no statistical differences in mCA clone size between participants with and without SGMs, regardless of SGM genes, variant allele fraction (VAF) ranges or number of SGMs (Supplementary Fig. 4A–C). Assessing the location of SGMs and mCAs demonstrated no overlaps for the location of SGMs and mCAs in the human genome (Supplementary Fig. 5, Supplementary Table 2).

Our analysis assessing whether mCAs occurred more frequently at older ages than SGMs also showed no significant difference between groups with SGMs compared to those with mCA (Supplementary Fig. 6). While we found no significant association between the occurrence of mCA and SGMs, an inverse association between mLOY with SGMs was previously reported, supporting the notion that haematopoietic stem cells may have a limited capacity to tolerate multiple types of genetic alterations driving CH^20,27^. This is consistent with the infrequent co-occurrence of mCAs with SGMs in our ARCHIVE cohort (Fig. 2B).

Building on our previous observations showing that SGMs are associated with elevated levels of inflammatory biomarkers^9^, we next examined whether mCAs alone or in combination with SGMs exhibited similar associations with haematological parameters and inflammatory markers. Across the whole cohort, we found the same levels of inflammatory biomarkers and blood cell counts in participants harbouring mCA alone and participants without any genetic alterations, whereas the level of interleukin-6 (IL-6) in participants harbouring SGMs alone was significantly higher than those without any genetic alterations (Supplementary Fig. 7A).

Among PWH (HIV-positive participants), we observed no differences between SGMs and mCA in their relationship with blood cell counts. However, participants with co-occurrence of SGMs and mCA exhibited a higher level of IL-6 compared to participants with mCA alone (Supplementary Fig. 7B); this was not observed in participants without HIV (Supplementary Fig. 7C). Interestingly, our analysis revealed an association between the risk of mCA or mLOY positivity and lower current or nadir CD4 levels in PWH (Fig. 2C, Supplementary Fig. 8), which was independent of SGM status, age and smoking history. This was consistent with the MACS cohort study, reporting higher mLOY frequency in PWH with lower CD4 + T cell counts^24^. However, we found no significant correlation between CD4 + T cell count and cell fraction of mCA or mLOY in ARCHIVE cohort (Supplementary Fig. 9).

Taken together, the increased risk of mCA among PWH with lower CD4 + T cell counts supports a hypothesis in which impaired immunosurveillance in HIV favours mCA persistence. This contrasts with the inflammatory environment driving SGM-driven CH previously reported in the same cohort^9^.

While we leveraged MoChA, a highly sensitive method for detecting mCA events at low cell fractions, given the lower genomic resolution of SNP array relative to whole genome sequencing data and the modest cohort size, small-scale events or those events from very minor cell clone may have remained undetected. Furthermore, given the predominance of male participants in ARCHIVE cohort, generalisability of our findings is likely greater for male PWH.

In summary, these results demonstrated for the first time that mCA and SGM-driven CH differ in their associations with HIV and inflammation, implying distinct immunological origins. Further elucidation of these mechanistic differences and their impact on comorbidities in PWH will be critical to inform future CH monitoring and intervention strategies.

## Methods

### Study population

The ARCHIVE study is a longitudinal cohort study (NCT04641013) evaluating biological aging and clinical outcomes in older adults with and without HIV. It enrolled 445 participants aged 55 years or older, including 219 PWH and 226 participants without HIV^9^. The research protocol, consent form and associated documents were approved by the St Vincent’s Hospital Human Research Ethics Committee in New South Wales, Australia. All individuals gave written informed consent to participate in the study. All ethical regulations relevant to human research participants were followed. Demographic and clinical data, such as age, gender, sexual orientation, most recent vital signs, medical history of diagnoses and the results of standard-of-care blood tests were obtained from participant medical records. For PWH, HIV disease duration, ART duration and the most recent CD4 count (i.e., current CD4) were also recorded. Participants also provided blood samples for full blood evaluation and inflammatory biomarker testing^9^.

### Whole blood sample processing and SGM detection

Whole blood samples were collected from participants at enrolment into the ARCHIVE study and QIAsymphony DNA Midi Kit (Qiagen, 931255) was used for DNA extraction according to the manufacturer’s protocols. Whole blood DNA was used for screening participants for SGM using a bespoke targeted deep sequencing (TS) amplicon panel designed across 55 genes recurrently mutated in haematological malignancies, described previously^9^.

### mCA detection

We processed 445 whole blood samples for SNP array genotyping by Infinium Global Screening Array (GSA) kit (Illumina) using 654,027 SNP probes (Fig. 1A). We utilised the Mosaic Chromosomal Alterations (MoChA) software and the pipeline (https://github.com/freeseek/mocha), which is optimised for calling mCAs from genotype data^10,11^. Throughout the analysis pipeline, GeneCall algorithm and gtc2vcf plugin from bcftools were used to transform raw intensity data of SNP probes to genotype data and generate file in VCF format. Variants with low genotyping quality, low divergence (3%), low estimated genotype calling rate (call_rate<0.97) and excess heterozygosity were filtered out prior to haplotype phasing with SHAPEIT4 software. Bcftools concat was used to concatenate phased genotypes into a single VCF file. Finally, MoChA caller was applied to detect mCAs using allele frequency (BAF) and Log R ratio (LRR) values and default parameter settings. MoChA pipeline excludes highly polymorphic regions, i.e., MHC (chr6: 27486711–33448264) and KIR (chr19: 54574747–55504099) from mCA calling. To exclude potential constitutional duplications and minimise false positive mCA events, we excluded mCA with length <2 Mb and relative coverage >2.5. Mosaic loss of chromosome Y (mLOY) events were identified by excluding mCAs on chromosome Y labelled as gain, undetermined and CN-LOH in the analysis output table. The major outputs of the analyses include fraction of cells harbouring mCA (cell fraction), size and type of mCA detected for each sample.

### Statistics and reproducibility

We used Prism 10.4.1 and R 4.4.2 for statistical analysis. Chi-squared test was used to assess the relationship between mCA and SGMs. Investigators analysing mCA and SGM data were blinded to participants’ HIV status. We employed Welch’s *t*-test between groups for cell fraction comparison after log transformation. Kruskal–Wallis test was used to compare blood cell counts and inflammatory markers of participants with different mutational statuses. Multivariate logistic regression was used to evaluate HIV and mCA relationship adjusting for the effects of other mCA-related variables including age, smoking history and SGM status.

### Reporting summary

Further information on research design is available in the Nature Portfolio Reporting Summary linked to this article.

## Supplementary information

Transparent Peer Review file

Supplementary Figs. and tables

Description of Additional Supplementary Files

Supplementary data 1

Reporting Summary

## Data Availability

The data underlying the figures in this study are provided as Supplementary Data 1. As the study is part of an ongoing clinical cohort study, any request for other raw or processed data and materials will undergo review by the corresponding authors to ensure compliance with ethical standards and study protocols. Access to patient-related data, particularly combined datasets that include clinical details, will require approval from the ARCHIVE study steering committee due to confidentiality considerations.

## Code availability

Data supporting the findings of this study can be obtained from the lead author upon request. This publication does not include original code. Further details necessary to reproduce the analyses presented in this work are available from the lead contact upon reasonable request.
