# Supplementary material for: Mosaic chromosomal alterations are suppressed in older adults with HIV
Source: Commun Biol. 2026 Jul 21;9:1001. doi: 10.1038/s42003-026-10532-1 (PMC13389083; doi:10.1038/s42003-026-10532-1)
Supplement: Supplementary file 2 — Supplementary Figs. and tables [file 42003_2026_10532_MOESM2_ESM.pdf]

Supplementary figures and tables

Mosaic chromosomal alterations are suppressed in older adults with HIV

Kiarash Behrouzfar[1] ✉, Win Min Han[2] ✉, Rashindrie Perera[3,4], Jason Li[4], Katherine E Scull[1], Kerryn Howlett[1], Mark Bloch[2,5], David A. Baker[6], Beng Eu[7,8], Ellen Bowden-Reid[2], Don E. Smith[9,10], Jennifer F. Hoy[11], Ian Woolley[1,11,12], Robert Finlayson[13], David J. Templeton[2,14,15], Gail V Matthews[2,16], Jane Costello[17], Mark A. Dawson[18,19,20], Sarah-Jane Dawson[18,19,20], Mark N. Polizzotto[2,21], Kathy Petoumenos[2], Nila J Dharan[2] ✉, Paul Yeh[1,22] ✉ * for the ARCHIVE Study Group±

1. Department of Medicine, School of Clinical Sciences at Monash Health, Monash University, Clayton, VIC, Australia
2. Kirby Institute, University of New South Wales, Sydney, NSW 2052, Australia
3. School of Electrical, Mechanical and Infrastructure Engineering, University of Melbourne, Melbourne, VIC 3010, Australia
4. Division of Cancer Research, Peter MacCallum Cancer Centre, Melbourne, VIC 3000, Australia
5. Holdsworth House Medical Practice, Sydney, NSW 2010, Australia
6. East Sydney Doctors, Sydney, NSW 2010, Australia
7. Prahran Market Clinic, Melbourne, VIC 3181, Australia
8. Melbourne Medical School, University of Melbourne
9. Albion Centre, South Eastern Sydney Local Health District, Sydney, NSW 2010, Australia
10. School of Population Health, University of New South Wales, Sydney, NSW 2033, Australia
11. Department of Infectious Diseases, School of Translational Medicine, The Alfred Hospital and Monash University, Melbourne, VIC 3004, Australia
12. Centre for Inflammatory Diseases, Monash University, Clayton, VIC 3168, Australia
13. Taylor Square Private Clinic, Darlinghurst, NSW 2010, Australia
14. Department of Sexual Health Medicine and Sexual Assault Medical Service, Sydney Local Health District, Sydney, NSW 2050, Australia
15. Discipline of Medicine, Central Clinical School, Faculty of Medicine and Health, The University of Sydney, Sydney, NSW 2050, Australia
16. St Vincent's Hospital, Darlinghurst, NSW 2010, Australia
17. Positive Life, Sydney, NSW 2010, Australia
18. Peter MacCallum Cancer Centre, Melbourne, VIC 3000, Australia
19. Sir Peter MacCallum Department of Oncology, University of Melbourne, Melbourne, VIC 3052, Australia
20. Collaborative Centre for Genomic Cancer Medicine, University of Melbourne, Melbourne, VIC 3052, Australia
21. Clinical Hub for Interventional Research, Australian National University, Canberra, ACT 2601, Australia
22. Monash Haematology, Monash Health, Clayton, VIC 3168, Australia

✉ These authors contributed equally.

***Correspondence:** Dr Paul Yeh (paul.yeh@monash.edu)

±Kiarash Behrouzfar[1], Win M Han[2], Rashindrie Perera[3,4], Jason Li[4], Katherine E Scull[1], Kerryn Howlett[1], Mark Bloch[2,5], Trina Vincent[5], David A. Baker[6], Beng Eu[7,8], Helen Lau[7], Ellen Bowden-Reid[2], Don E. Smith[9,10], Kathryn Acklom[9], Jennifer F. Hoy[11], Sally Price[11], Ian Woolley[1,11,12], Jessica O'Bryan[11], Robert Finlayson[13], David J. Templeton[2,14,15], Brett Sinclair[14], Gail V Matthews[2,16], Jane

Costello[17], Mark A. Dawson[18,19,20], Sarah-Jane Dawson[18,19,20], Mark N. Polizzotto[2,21], Kathy Petoumenos[2], Nila J. Dharan[2] and Paul Yeh[1,22]

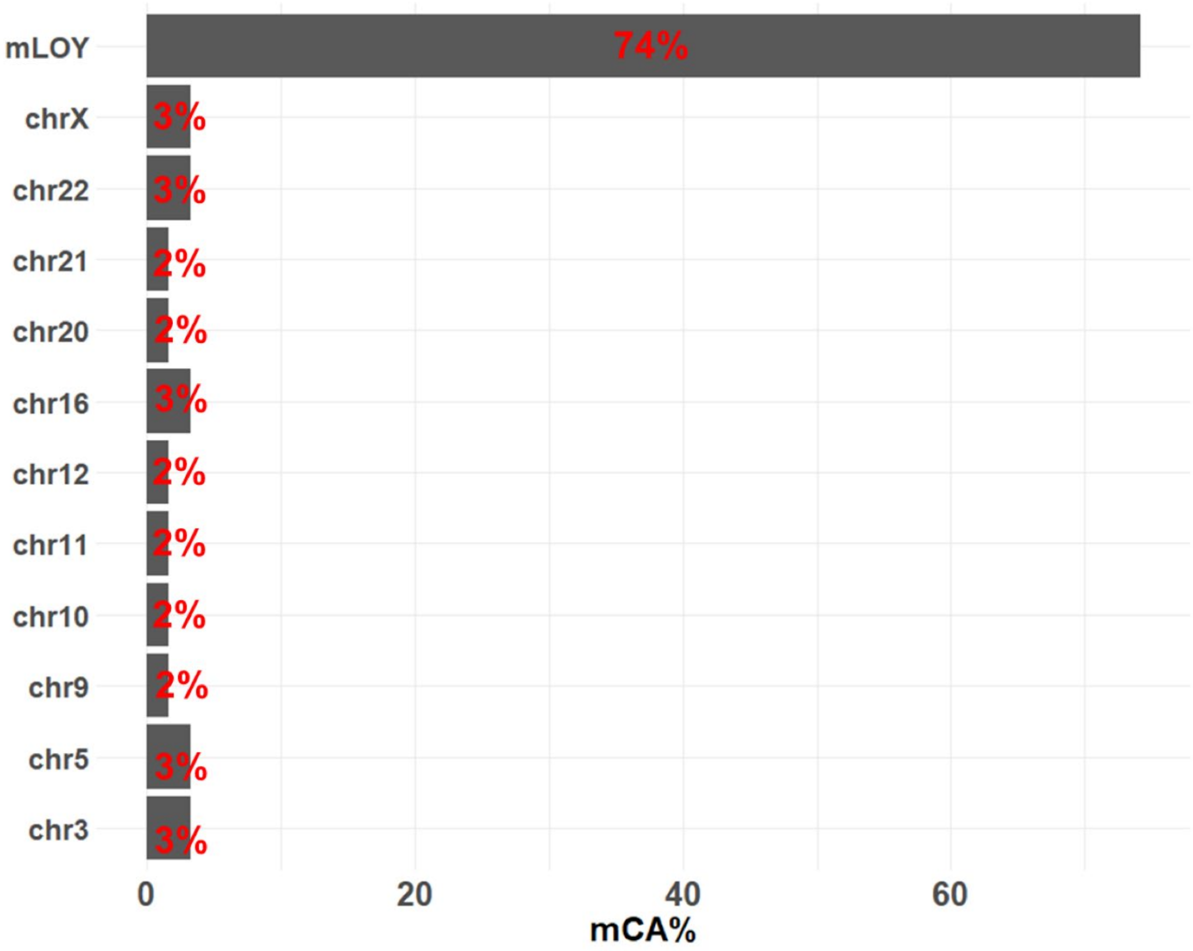

Supplementary figure 1. Distribution of mosaic chromosomal alterations (mCA) events across chromosomes. Red numbers inside bars correspond to the percentage of mCA events for each chromosome.

A.

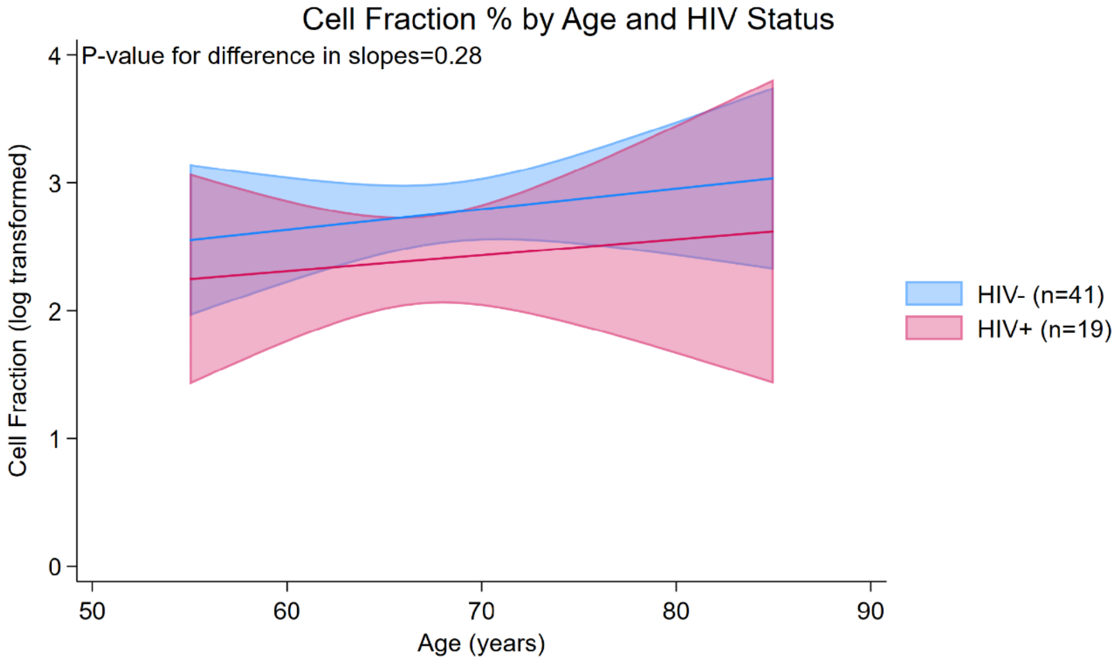

B.

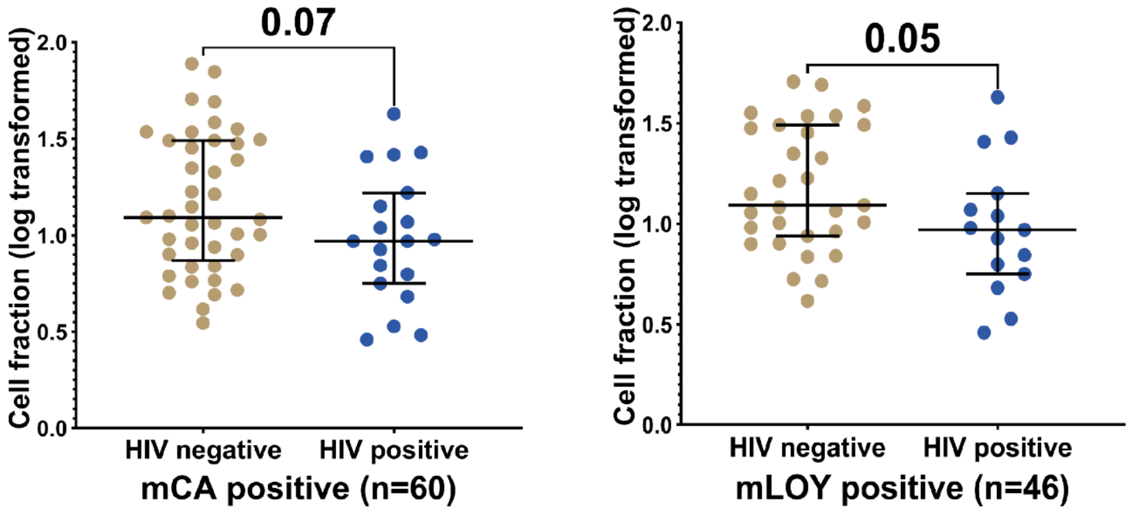

Supplementary figure 2. Clone size (cell fraction) comparison of cells harbouring mosaic Chromosomal Alterations (mCA) between participants with and without HIV. A. Cell fraction % (log transformed) over increasing age, by HIV status. Mean values are represented by the regression lines, and the shaded areas represent upper and lower 95% CIs. The confidence limits were obtained from a linear regression model with main effects and an interaction term for age and HIV status (P-interaction=0.28). **B.** Mean comparison of cell fraction % (log transformed) between groups of participants with and without HIV. P values of t.test are shown for each comparison.

A.

All participants (n=445)		SGM status		Chi-squared P value
	**Total**	**Negative**	**Positive**	
**mCA status**				**0.90**
Negative	385 (86.5)	299 (86.4)	86 (86.9)	
Positive	60 (13.5)	47 (13.6)	13 (13.1)	

Male participants (n=428)		SGM status		Chi-squared P value
	**Total**	**Negative**	**Positive**	
**mLOY status**				**0.93**
Negative	382 (89.2)	297 (89.2)	85 (89.5)	
Positive	46 (9.8)	36 (10.8)	10 (10.5)	

B.

HIV-positive (n=219)		SGM status		Chi-squared P value
	**Total**	**Negative**	**Positive**	
**mCA status**				**0.360**
Negative	200 (91.3)	146 (92.4)	54 (88.5)	
Positive	19 (8.7)	12 (7.6)	7 (11.5)	

HIV-positive (n=215)		SGM status		Chi-squared P value
	**Total**	**Negative**	**Positive**	
**mLOY status**				**0.596**
Negative	200 (93.0)	146 (93.6)	54 (91.5)	
Positive	15 (7.0)	10 (6.4)	5 (8.5)	

C.

HIV-negative (n=226)		SGM status		Chi-squared P value
	**Total**	**Negative**	**Positive**	
**mCA status**				**0.680**
Negative	185 (81.9)	153 (81.4)	32 (84.2)	
Positive	41 (18.1)	35 (18.6)	6 (15.8)	

HIV-negative (n=213)		SGM status		Chi-squared P value
	**Total**	**Negative**	**Positive**	
**mLOY status**				**0.901**
Negative	182 (85.5)	151 (85.3)	31 (86.1)	
Positive	31 (14.5)	26 (14.7)	5 (13.9)	

Supplementary figure 3. Mosaic chromosomal alterations (mCA) prevalence comparison between participants with and without somatic gene mutations (SGM). A. Contingency table demonstrating the distribution of mCA and or mosaic loss of chromosome Y (mLOY) events detected in all participants, **B.** People with HIV; PWH (HIV-positive) and **C.** participants without HIV (HIV-negative) with and without SGMs. Rows and column represent the presence or absence of mCA and SGMs, respectively. Cell values indicate the number of participants, with percentages in parentheses representing the column-wise percentage of each event category. Chi-squared test was used to assess the relationship between mCA and SGMs.

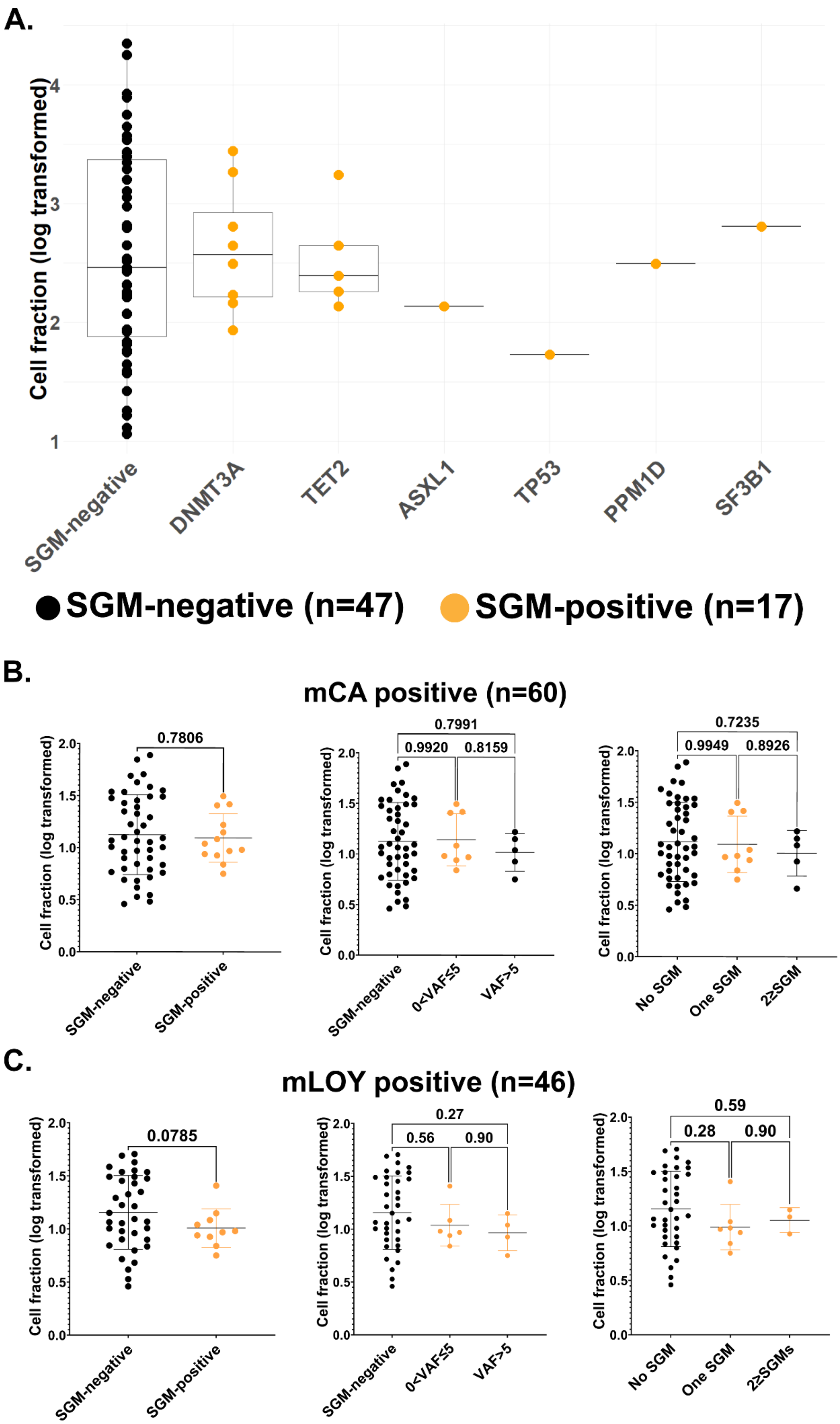
A.
Cell fraction (log transformed)
SGM-negative
DNMT3A
TET2
ASXL1
TP53
PPM1D
SF3B1
SGM-negative (n=47)
SGM-positive (n=17)
B.
mCA positive (n=60)
0.7806
0.7991
0.9920
0.8159
0.7235
0.9949
0.8926
SGM-negative
SGM-positive
0<VAF≤5
VAF>5
No SGM
One SGM
2≥SGM
C.
mLOY positive (n=46)
0.0785
0.27
0.56
0.90
0.59
0.28
0.90
2≥SGMs

Supplementary figure 4. Mosaic chromosomal alterations (mCA) clone size comparison between participants with and without somatic gene mutations (SGMs). A. mCA clone size (log transformed cell fraction%) comparison between participants harbouring SGMs stratified by gene (orange) and participants without SGMs (black). Welch's t-test was used to compare cell fraction of groups with at least 3 events (i.e. *DNMT3A and TET2*) and SGM-negative group. **B.** Log transformed cell fraction (cf)% comparison of mCA and **C.** mLOY between SGM-negative and SGM-positive participants, stratified by different ranges of variant allele frequency (VAF) for CH mutations and stratified by the number of mutations. P values for Welch t-test between groups with different SGM statuses are shown above each pair of comparison.

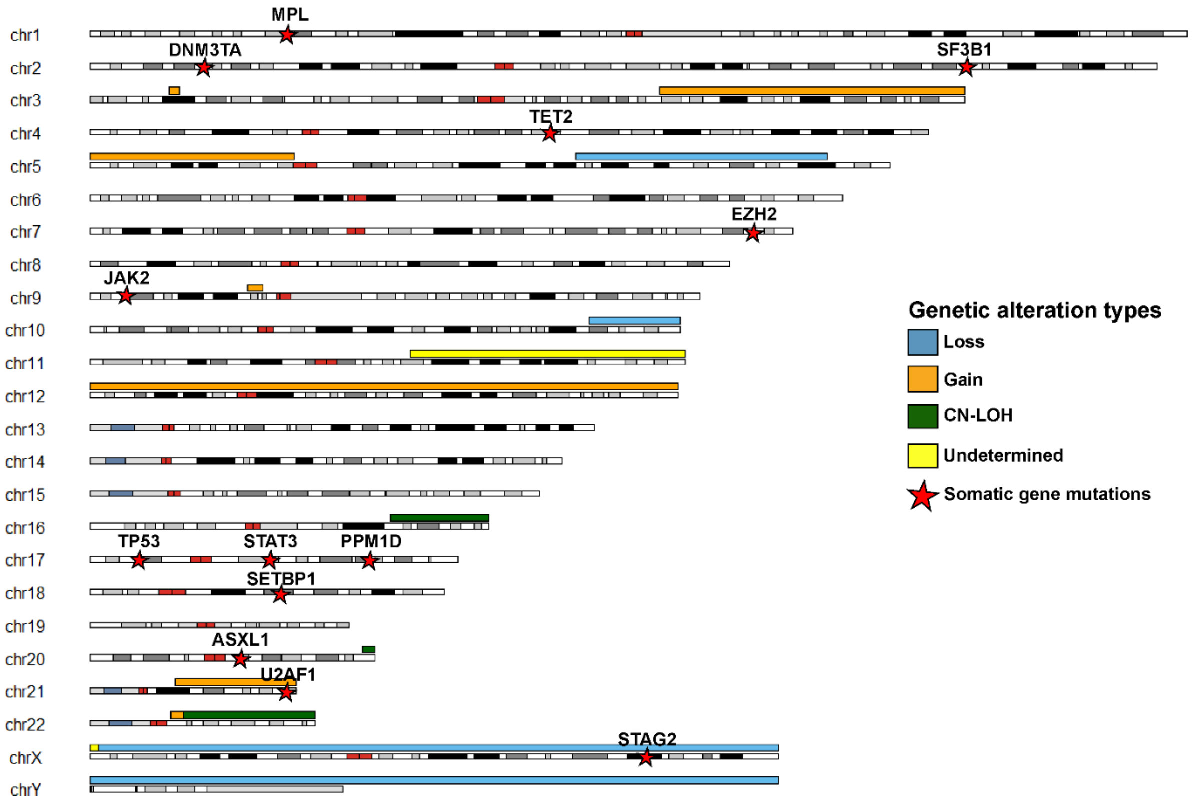

Supplementary figure 5. Schematic representation of the location and types of mosaic chromosomal alterations (mCA) and somatic gene mutations (SGMs). mCA types are Gain (orange), Loss (lightblue), Copy number-loss of heterozygosity (CN-LOH, darkgreen) and Undetermined (yellow).

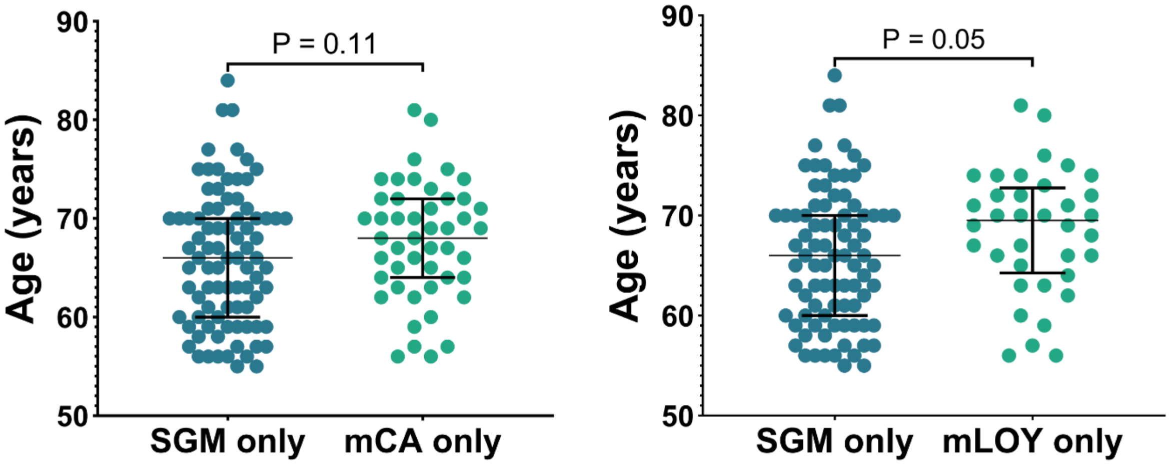

Supplementary figure 6. Mean age comparison between participants with mosaic chromosomal alterations (mCA) or mosaic loss of chromosome Y (mLOY) alone (mCA and mLOY only) and individuals harbouring somatic gene mutations (SGMs) alone (SGM only).

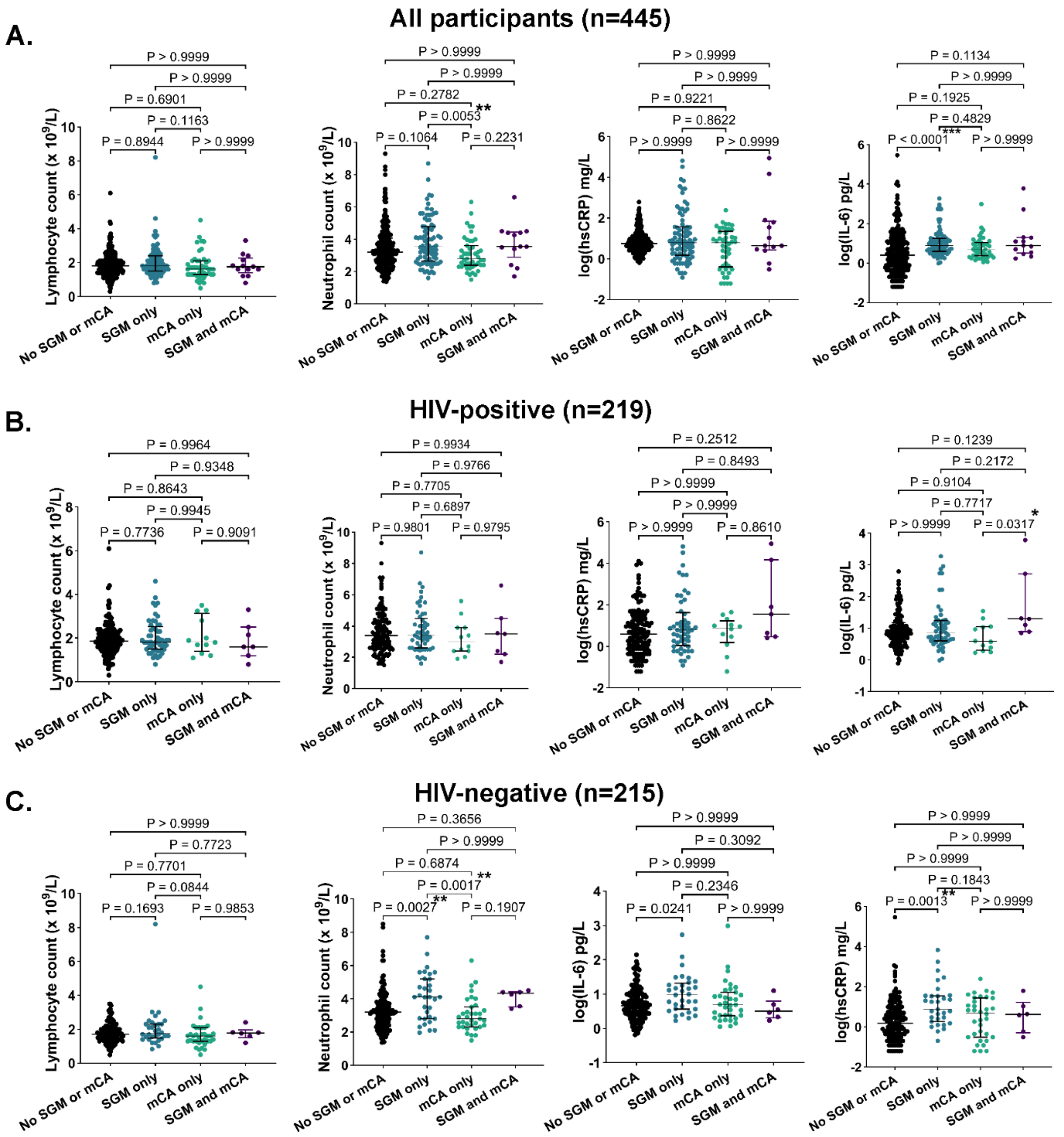

Supplementary figure 7. Lymphocyte and Neutrophile counts comparison between individuals with different mutational statuses. A. Graphs show median and interquartile ranges of neutrophils and lymphocyte counts for comparing all participants, **B.** People with HIV; PWH (HIV-positive) and **C.** Participants without HIV (HIV-negative) with different mutational statuses. Kruskall-wallis test was used for the overall group comparison and followed by Dunn's test for multiple testing assessment. Pairwise adjusted p-values are shown above each comparison. Two-sided p-values were corrected for multiple comparisons within each subgroup using the Benjamini-Hochberg false discovery rate procedure. BH-FDR adjusted overall p-values for all participants were: log(IL-6) p=0.0001, WBC p=0.008, neutrophil p=0.008, log(hsCRP) p=0.168, lymphocyte p=0.428; for HIV-positive individuals: log(IL-6) p=0.238, WBC p=0.889, neutrophil p=0.889, log(hsCRP) p=0.403, lymphocyte p=0.889; and for HIV-negative

individuals: log(IL-6) p=0.033, WBC p=0.001, neutrophil p=0.001, log(hsCRP) p=0.005, lymphocyte p=0.155.* P value< 0.05, ** P values<0.01 and *** P values <0.001.

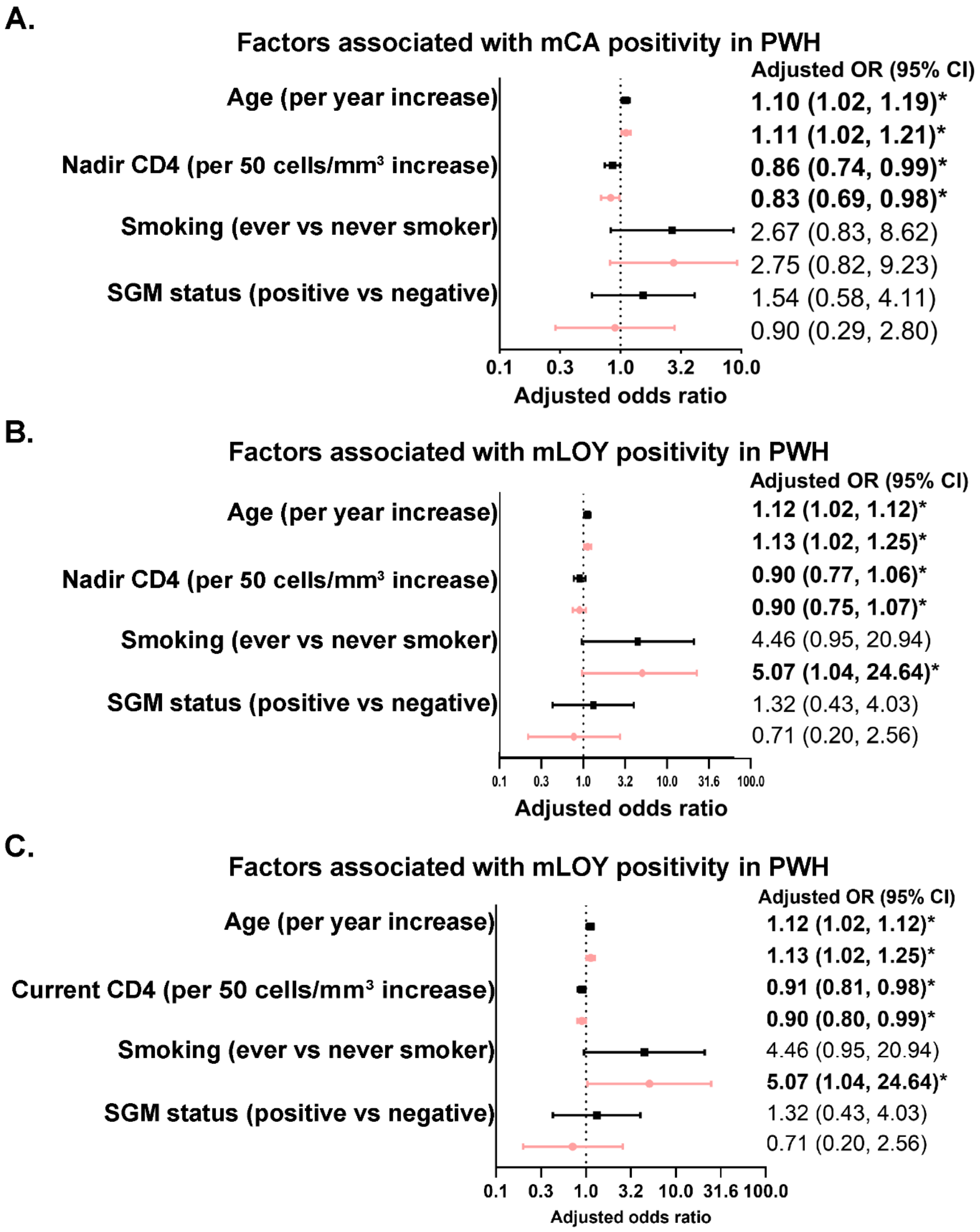

Supplementary figure 8. The relationship of clinical and demographic variables with mosaic chromosomal alterations (mCAs) and mosaic loss of chromosome Y (mLOY) in people with HIV (PWH). Forest plot shows the outputs of univariate and multivariate logistic regression analysis to assess the relationship between **A.** mCA or **B & C.** mLOY and clinical data including age, lowest CD4+ T cells (nadir CD4), current CD4+ T cells, smoking history and somatic gene mutation (SGM) status.

The mean odds ratio and 95% confidence interval values are shown in the right-end column of the table.
* P-value < 0.05, ** P-value < 0.01 and *** P-value < 0.001.

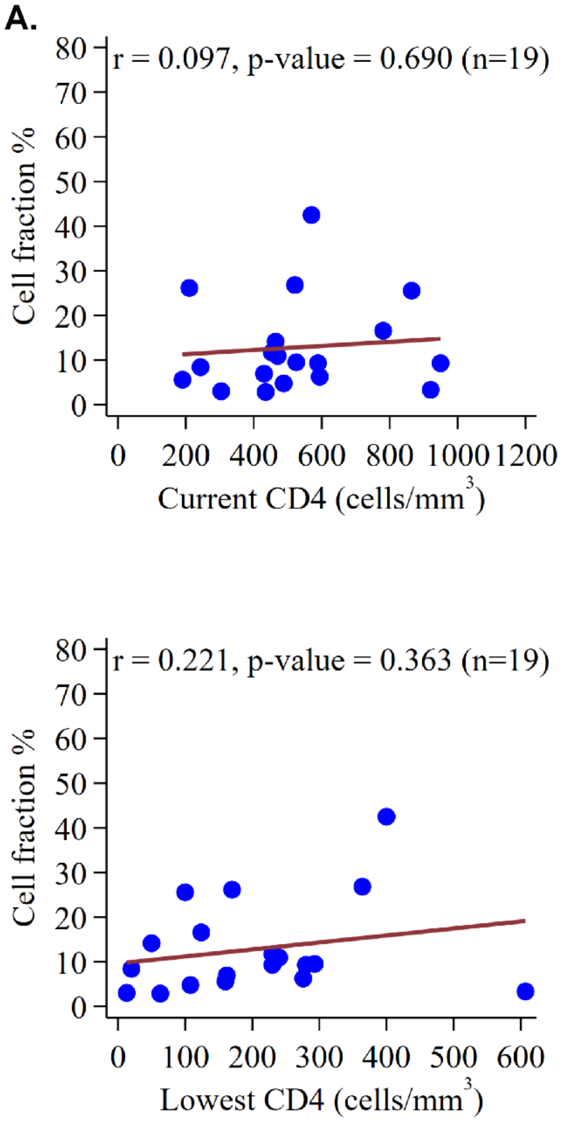

Supplementary figure 9. The Pearson correlation plot of cell fraction (%) of mosaic chromosomal alterations (mCA) and mosaic loss of chromosome Y (mLOY) with Current and Nadir CD4+ T cell counts in people with HIV (PWH).

Supplementary Table 1. Distribution of demographic and clinical characteristics of individuals with and without mosaic chromosomal alterations (mCAs) and mosaic loss of chromosome Y (mLOY) in the ARCHIVE cohort.

Characteristics	mCA positive (n=60)	mCA negative (n=385)	mLOY positive (n=46)	mLOY negative (n=382)	Total (n=445)
**Age (years)**					
Median (IQR)	69 (64-72)	63 (59-68)	69 (64-73)	63 (59-68)	63 (59-69)
55-64 (%)	17 (28.3)	226 (58.7)	12 (26.1)	223 (58.4)	243 (54.6)
65-74 (%)	37 (61.7)	140 (36.4)	29 (63.0)	141 (36.9)	177 (39.8)
≥75 (%)	6 (10)	19 (4.9)	5 (10.9)	18 (4.7)	25 (5.6)
**Gender**					
Male (%)	58 (96.7)	370 (96.1)	46 (100)	382 (100)	428 (96.2)
Female (%)	2 (3.3)	15 (3.9)	0 (0)	0 (0)	17 (3.8)
**Sexual orientation**					
MSM (%)	50 (83.3)	339 (88.1)	38 (82.6)	351 (91.9)	389 (87.4)
Other (%)	9 (15.0)	34 (8.8)	7 (15.2)	20 (5.2)	43 (9.7)
Missing (%)	1 (1.7)	12 (3.1)	1 (2.2)	11 (2.9)	13 (2.9)
**Smoking**					
Never smoked (%)	22 (36.7)	172 (44.7)	14 (30.4)	174 (45.5)	194 (43.6)
Past smoking (%)	23 (38.3)	131 (34.0)	20 (43.5)	129 (33.8)	154 (34.6)
Current smoking (%)	8 (13.3)	38 (9.7)	7 (15.2)	34 (8.9)	46 (10.3)
Missing (%)	7 (11.7)	44 (11.4)	5 (10.9)	45 (11.8)	51 (11.5)
**Alcohol consumption**					
Never drank (%)	0 (0)	17 (4.4)	0 (0)	16 (4.2)	17 (3.8)
Past drinking (%)	11 (18.3)	61 (15.8)	7 (15.2)	58 (15.2)	72 (16.2)
Current drinking (%)	42 (70.0)	263 (68.3)	34 (73.9)	263 (68.9)	305 (68.5)
Missing (%)	7 (11.7)	44 (11.4)	5 (10.9)	45 (11.8)	51 (11.5)
**HIV status**					
Negative (%)	41 (68.3)	185 (48.1)	31 (67.4)	182 (47.6)	226 (50.8)
Positive (%)	19 (31.7)	200 (51.9)	15 (32.6)	200 (52.4)	219 (49.2)
**Viral hepatitis B** (%)	3 (5.0)	28 (7.3)	3 (6.5)	27 (7.1)	31 (6.7)
**Viral hepatitis C** (%)	7 (11.7)	33 (8.6)	6 (13.0)	30 (7.9)	40 (9.0)

Supplementary Table 2. Distribution of mosaic Chromosomal Alterations (mCA) types across HIV-positive and HIV-negative. Letters in the parentheses refer to the location of mCA events on q or p arm of the chromosome.

Chromosomes	HIV-negative				HIV-positive				
	Loss	Gain	CN-LOH	Undetermined	Loss	Gain	CN-LOH	Undetermined	Co-occurrence with SGM
**Chr1**	0	0	0	0	0	0	0	0	-
**Chr2**	0	0	0	0	0	0	0	0	-
**Chr3**	0	2 (q, p)	0	0	0	0	0	0	-
**Chr4**	0	0	0	0	0	0	0	0	-
**Chr5**	1 (p)	0	0	0	0	1 (q)	0	0	-
**Chr6**	0	0	0	0	0	0	0	0	-
**Chr7**	0	0	0	0	0	0	0	0	-
**Chr8**	0	0	0	0	0	0	0	0	-
**Chr9**	0	1 (p)	0	0	0	0	0	0	-
**Chr10**	1(q)	0	0	0	0	0	0	0	-
**Chr11**	0	0	0	1 (q)	0	0	0	0	-
**Chr12**	0	1(p, q)	0	0	0	0	0	0	-
**Chr13**	0	0	0	0	0	0	0	0	-
**Chr14**	0	0	0	0	0	0	0	0	-
**Chr15**	0	0	0	0	0	0	0	0	-
**Chr16**	0	0	0	0	1 (q)	0	1 (q)	0	1
**Chr17**	0	0	0	0	0	0	0	0	-
**Chr18**	0	0	0	0	0	0	0	0	-
**Chr19**	0	0	0	0	0	0	0	0	-
**Chr20**	0	0	0	0	0	0	1 (q)	0	1
**Chr21**	0	1 (q)	0	0	0	0	0	0	-
**Chr22**	0	2 (q)	0	0	0	0	0	0	1
**ChrX**	0	1 (p,q)	0	1 (p,q)	0	0	0	0	-
**ChrY**	31 (p,q)	0	0	0	15 (p,q)	0	0	0	10
