# Supplementary material for: Mosaic chromosomal alterations are suppressed in older adults with HIV
Source: Commun Biol. 2026 Jul 21;9:1001. doi: 10.1038/s42003-026-10532-1 (PMC13389083; doi:10.1038/s42003-026-10532-1)
Supplement: Supplementary file 3 — Description of Additional Supplementary Files [file 42003_2026_10532_MOESM3_ESM.pdf]

Description of Additional Supplementary files

File name: Supplementary Data 1

Description: The source numerical data of graphs in the manuscript
