# Supplementary material for: Mosaic chromosomal alterations are suppressed in older adults with HIV
Source: Commun Biol. 2026 Jul 21;9:1001. doi: 10.1038/s42003-026-10532-1 (PMC13389083; doi:10.1038/s42003-026-10532-1)
Supplement: Supplementary file 1 — Transparent Peer Review file [file 42003_2026_10532_MOESM1_ESM.pdf]

nature portfolio

Peer Review File

Mosaic chromosomal alterations are suppressed in older adults with HIV

Corresponding Author: Dr Paul Yeh

This file contains all reviewer reports in order by version, followed by all author rebuttals in order by version.

Version 0:

Reviewer comments:

Reviewer #1

(Remarks to the Author)
Congratulations to the authors on an excellent paper. The text and figures are of outstanding quality, and this is clearly an important and impactful piece of work. I have enjoyed reviewing it. Here are my suggestions for your consideration:

The study raises important questions but has several conceptual and methodological limitations. It is unclear whether the authors explored the association between HIV status and mCA type (deletion, duplication, C-OH), or examined the specific genomic regions affected by mCAs, particularly those unique to people with HIV (that would be interesting, however, the cohort is pretty small).
From a statistical perspective, the covariates included in the multivariate logistic regression models are not explicitly specified in the Methods section, limiting transparency, interpretability, and assessment of confounding, in addition it is unclear whether correction for multiple comparisons was applied.
Some important limitations of the analysis should be acknowledged in the manuscript, including that mCAs were detected using a 654,027-SNP array rather than whole-genome sequencing. Lower SNP density reduces the ability to detect small-segment aberrations, often requiring a minimum mCA size of 1-2 Mb for reliable detection. Additionally, the ARCHIVE cohort is predominantly male and older (≥55 years), and participants are engaged in clinical care, introducing potential selection and survivor bias and limiting generalizability to women, younger individuals, and broader HIV populations.

Reviewer #2

(Remarks to the Author)
This is an impressive add-on to the basic ARCHIVE study of the effects of aging on PWH. It does a deep dive into the effect of mCA in contrast to SGMs. It tries to relate this information to the relative risk of age-related comorbidities. The microbiological part of this paper is well-structured and the types of analytic tools employed (MoChA, etc.) are well described although this paper does not evaluate whether the tools actually measure what they say they measure. A bit more skepticism would be in order here. The authors statistical analysis sticks to basic measures of differences in means between groups but employs them correctly.

Where the paper needs more work is in relating their findings about SGMs vs. mCAs more specifically to the risks of contracting one of the diseases mentioned in the paper's introduction. Also, CD4+ counts only comes into play when assessing mLOY frequency. Since CD4+ cells are the targets of HIV, their relationship to the other factors under analysis should be more thorough.

Overall, this is an impressive addition to the ARCHIVE work to date.

Version 1:

Reviewer comments:

Reviewer #1

(Remarks to the Author)

All comments have been taken into account. I recommend this manuscript for publication.

Reviewer #2

(Remarks to the Author)
This revised version answers my previous comments. Again, my congratulations on an excellent study.

Open Access This Peer Review File is licensed under a Creative Commons Attribution 4.0 International License, which permits use, sharing, adaptation, distribution and reproduction in any medium or format, as long as you give appropriate credit to the original author(s) and the source, provide a link to the Creative Commons license, and indicate if changes were made.
In cases where reviewers are anonymous, credit should be given to 'Anonymous Referee' and the source.
The images or other third party material in this Peer Review File are included in the article’s Creative Commons license, unless indicated otherwise in a credit line to the material. If material is not included in the article’s Creative Commons license and your intended use is not permitted by statutory regulation or exceeds the permitted use, you will need to obtain permission directly from the copyright holder.
To view a copy of this license, visit https://creativecommons.org/licenses/by/4.0/

Reviewers' comments:

Reviewer #1 (Remarks to the Author):

1- Congratulations to the authors on an excellent paper. The text and figures are of outstanding quality, and this is clearly an important and impactful piece of work. I have enjoyed reviewing it. Here are my suggestions for your consideration:
The study raises important questions but has several conceptual and methodological limitations. It is unclear whether the authors explored the association between HIV status and mCA type (deletion, duplication, C-OH), or examined the specific genomic regions affected by mCAs, particularly those unique to people with HIV (that would be interesting; however, the cohort is pretty small).

Thank you and appreciate the reviewer's comments on the impact of study and the quality of figures. We agreed on the reviewer's comments on methodological limitations of our analysis and attempted to further clarify those limitations as well as the analysis method in the main text (line 159-164). We also added a supplementary table (supplementary table 2) demonstrating the number of events with their location and types for all participants, HIV-positive and HIV-negative participants to better specify the genomic location affected by mCA. Whilst there were differences in mCA type between groups, the small sample size limited meaningful interpretation, as noted by the reviewer.

We have reviewed other HIV cohorts to evaluate whether our events were also detected in other HIV cohorts. Interestingly, mLOY, chr5q loss and chr21q gain were also reported in a recent preprint from the Swiss HIV Cohort Study, Doi: https://doi.org/10.64898/2025.12.17.25342413 (line 95-96).

2- From a statistical perspective, the covariates included in the multivariate logistic regression models are not explicitly specified in the Methods section, limiting transparency, interpretability, and assessment of confounding, in addition it is unclear whether correction for multiple comparisons was applied.

We have now explicitly specified the covariates included in the multivariate logistic regression models in the Methods section (line 211) to improve transparency and interpretability. Regarding multiple comparisons, we wish to clarify that the p-values displayed in the figures represent adjusted p-values from Dunn's post-hoc test following the Kruskal-Wallis test, which corrects for multiple pairwise comparisons within each variable. To further address the concern regarding testing multiple variables simultaneously, we additionally applied the Benjamini-Hochberg false discovery rate (BH-FDR) procedure across all variables tested within each subgroup (all participants, HIV-positive, and HIV-negative). Our key findings remained consistent before and after BH-FDR correction, supporting the robustness of our results. The BH-FDR adjusted overall p-values have been incorporated into the figure caption of Supplementary Figure 7 for transparency.

3- Some important limitations of the analysis should be acknowledged in the manuscript, including that mCAs were detected using a 654,027-SNP array rather than whole-genome sequencing. Lower SNP density reduces the ability to detect small-segment aberrations, often requiring a minimum mCA size of 1-2 Mb for reliable detection. Additionally, the ARCHIVE cohort is predominantly male and older (≥55 years), and participants are engaged in clinical care, introducing potential selection and survivor bias and limiting generalizability to women, younger individuals, and broader HIV populations.

We thank the reviewer for highlighting the limitations of the study. We have added a paragraph, acknowledging the more restricted resolution of SNP array data to detect chromosomal alterations in comparison to whole-genome sequencing data. In addition, we acknowledged the limited generalizability of our results to the broader HIV populations (line 159-164).

Reviewer #2 (Remarks to the Author):

1- This is an impressive add-on to the basic ARCHIVE study of the effects of aging on PWH. It does a deep dive into the effect of mCA in contrast to SGMs. It tries to relate this information to the relative risk of age-related comorbidities. The microbiological part of this paper is well-structured and the types of analytic tools employed (MoChA, etc.) are well described although this paper does not evaluate whether the tools actually measure what they say they measure. A bit more skepticism would be in order here. The authors statistical analysis sticks to basic measures of differences in means between groups but employs them correctly.

We sincerely appreciate the reviewer's comments on our MoChA results. We have added a paragraph acknowledging the limitations in this method (line 159-164); however, it is worth mentioning that our results include several mCA calls such as chr5q loss and chr21q gain that were previously detected in other PWH cohorts (line 95-96, supplementary figure 5, supplementary table 2 were added).

2- Where the paper needs more work is in relating their findings about SGMs vs. mCAs more specifically to the risks of contracting one of the diseases mentioned in the paper's introduction. Also, CD4+ counts only comes into play when assessing mLOY frequency. Since CD4+ cells are the targets of HIV, their relationship to the other factors under analysis should be more thorough. Overall, this is an impressive addition to the ARCHIVE work to date.

We thank the reviewer for raising the point regarding the relationship between mCA or SGMs and the risks of developing comorbidities in PWH. This is indeed one of the main aims of ARCHIVE study and this part of study is still ongoing for both SGMs and mCAs in HIV-positive participants of ARCHIVE cohort. Furthermore, the association of some mCA calls detected in this study with the risks of haematological malignancies and cardiovascular diseases were previously reported in other studies. We added a paragraph describing those mCA calls and their link with comorbidities (line 97-101).
To address your comment on the relationship between CD4+ T cells and mLOY frequency, we

have also added two supplementary figures (supplementary figure 8 and 9). These graphs showed the significant correlation between the presence of mLOY and the most recent measurements of CD4+ T cell count before sample collection, although there was no significant correlation between mCA, mLOY cell fraction and lower CD4+ T cell count. This may suggest the potential link between mLOY and mCA with impaired immunosurveillance in HIV, however this is required to be assessed and validated in future studies.
